# Reply

**DOI:** 10.1016/j.xops.2023.100377

**Published:** 2023-08-03

**Authors:** Bethany E. Higgins, Giovanni Montesano, David M. Wright, Ruth E. Hogg, David P. Crabb

**Affiliations:** 1 Optometry and Vision Sciences; City, University of London, London UK; 2 Centre for Public Health, Queen's University Belfast, Belfast, UK

We thank Swain et al for their interest in our work^1^ and for the chance to address some perceived statistical issues. Their main criticism pertains to our use of parametric survival analysis to estimate the parameters of a model describing changes in Rod-intercept time (RIT) because of age and disease stage, which they define “questionable.” In their interpretation of the results, they explain how our model describes the changes in the hazard rate of reaching the rod-intercept among groups. This is certainly valid because the RIT effectively describes an “event” that is absolutely compatible with what is normally modeled with survival analysis methods. However, hazard rates are far from being the only correct interpretation of the results of parametric survival models.

Perhaps, Swain et al are not familiar with the well-established use of survival analysis methodology to estimate the parameters of a parametric model in the presence of censored data. Such data can arise, for example, from the limited dynamic range of some instruments, a common issue in psychophysical measures,^2^^,^^3^ including RIT, where measurements usually do not extend beyond a predetermined duration. For example, the *AER* package for R (R Foundation for Statistical Computing) can fit censored regression models (sometimes called Tobit models) with a variety of distributions (including Weibull) by simply being a convenient frontend for the *survival* package.^4^ The results obtained with the *AER* and *survival* packages are, in fact, identical. Another approach would be to use Bayesian computation, which can easily incorporate complex distributions for the response variable and account for censoring. This has been extensively used by our group in the field of perimetry^3^ and is identical to the modeling that would be performed for Bayesian survival analyses. Note that these models are mainly concerned with estimating how the parameters of the distribution of choice are influenced by variables of interest, like in standard regression models, without necessarily interpreting that distribution as a description of a hazard rate. These parameters can be used to make inferences about other descriptors of the modeled distribution, such as the median. This was extensively detailed in a previous publication for this specific application^2^ and is hopefully further clarified by the example in Figure 1.

**Figure 1:**
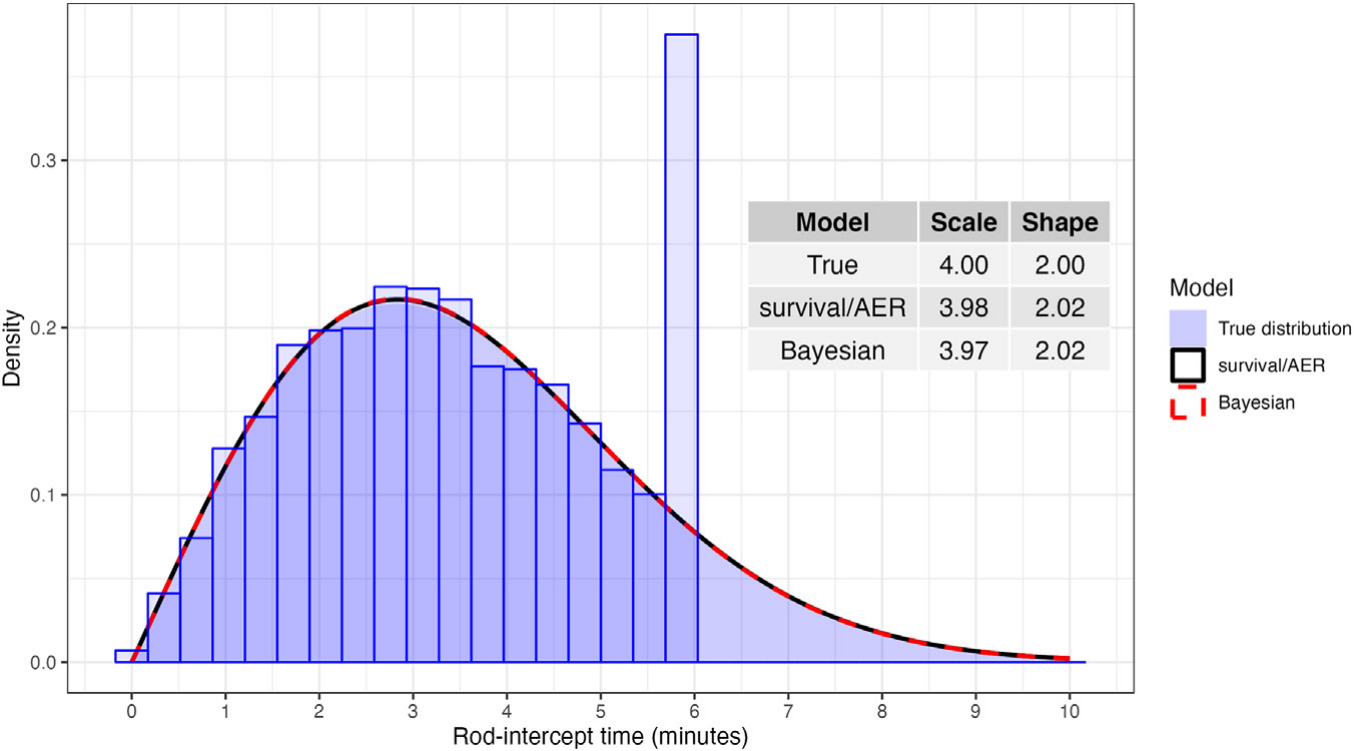
Censored rod-intercept time data (histogram) simulated from a Weibull distribution (shaded blue curve). The red and black curves are estimated from the censored data using the *survival* or *AER* package in R (same results, red) or Bayesian computation (black). The estimated and true scale and shape parameters for the Weibull distribution are reported in the table inset.

Certainly, we could have used the framework of censored regression to present our results. However, we wanted to take the chance to help educate readers about the methodology underlying censored models by using the familiar context and graphical tools of survival analysis. We are sorry this was confusing.

Finally, because our parametric modeling can incorporate the effect of covariates, it can model the changes in the distribution of RIT due, for example, to age. As correctly pointed out by the Swain et al, this means correcting the estimate, not the measured RIT value. We should have made clearer. However, this is also very different from modeling the change in odds ratio of having an “abnormal” RIT, because our approach would model the RIT as a continuous value rather than relying on a “normative” cut-off. We think this has additional merit.

In conclusion, we think statistical tools offer methods to estimate parameters of interest. The interpretation of these parameters should be provided by the researchers based on their knowledge of the phenomenon under investigation and, obviously, of the methodology adopted for the analysis. We also think it is unreasonable to restrict a survival analysis methods to the very specific applications for which they were originally designed, ignoring their ability to provide effective description of many other phenomena.

## References

[1] Higgins B.E., Montesano G., Crabb D.P. (2022). Assessment of the classification of age-related macular degeneration severity from the northern ireland sensory ageing study using a measure of dark adaptation. Ophthalmol Sci.

[2] Higgins B.E., Montesano G., Binns A.M., Crabb D.P. (2021). Optimising assessment of dark adaptation data using time to event analysis. Sci Rep.

[3] Montesano G., Garway-Heath D.F., Ometto G., Crabb D.P. (2021). Hierarchical censored Bayesian analysis of visual field progression. Transl Vis Sci Technol.

[4] Kleiber C., Zeileis A. (2008).

